# Prospective phase II clinical trial of molecular glioblastoma (historical grade 2 and 3 IDH wildtype gliomas) preliminary novel exploratory analyses: Treatment intensification, margin reduction and epigenetic stratified outcomes with radiation therapy and chemotherapy

**DOI:** 10.1007/s11060-025-05269-6

**Published:** 2025-11-05

**Authors:** Debra Nana Yeboa, Benjamin T. Whitfield, Ruitao Lin, Chinenye Lynette Ejezie, Todd A. Swanson, Thomas H. Beckham, Chenyang Wang, Brian De, Subha Perni, Martin C. Tom, Jing Li, Susan L. McGovern, Rebecca Harrison, Nazanin K. Majd, Vinay K. Puduvalli, Ashley E. Aaroe, Monica Loghin, Barbara J. O’Brien, Anuj D. Patel, Chirag B. Patel, Jeffrey S. Wefel, Ceylan Altintas Taslicay, Maria Gule-Monroe, Arnold C. Paulino, Mary Frances McAleer, David R. Grosshans, Amol J. Ghia, Wen Jiang, Caroline Chung, Moshe Maor, Cheng-Han Yang, Maria A. Gubbiotti, Carlos Kamiya-Matsuoka, Leomar Y. Ballester, Shiao-Pei Weathers, Jason T. Huse

**Affiliations:** 1 Department of Radiation Oncology, University of Texas MD Anderson Cancer Center, Houston, TX USA; 2 Departments of Pathology and Translational Molecular Pathology, University of Texas MD Anderson Cancer Center, Houston, TX USA; 3 Department of Biostatistics, Yale University, New Haven, CT USA; 4 Department of Neuro-Oncology, University of Texas MD Anderson Cancer Center, Houston, TX USA; 5 Department of Neuroradiology, University of Texas MD Anderson Cancer Center, Houston, TX USA; 6 Quantitative Imaging Analysis Core, University of Texas MD Anderson Cancer Center, Houston, TX USA; 7 Division of Neurology, University of British Columbia, Vancouver, Canada; 8 1515 Holcombe Blvd, Unit 97, Houston, TX 77030 USA; 9 Department of Health Sciences, Towson University, Towson, MD USA

**Keywords:** Molecular glioblastoma, Molecular WHO grade 4 glioma, Non-enhancing, Survival, Chemoradiotherapy

## Abstract

**Purpose:**

Molecular glioblastoma (molGBM) is a variant lacking the full histopathological profile of glioblastoma. We report a trial aimed at addressing the optimal management of this newly recognized rarer form of glioma.

**Methods:**

In this phase II study, molGBM patients were treated with radiation to a dose of 60Gy to the gross tumor volume (GTV) only, and a single smaller margin potentially as low as 1cm to the clinical tumor volume (CTV). As the trial is ongoing, we report on important exploratory biomarker findings correlating with median overall survival (mOS). Analysis included Kaplan-Meier and univariable/multivariable cox proportional hazard models. Available pre-operative tissue was subjected to epigenetic/DNA methylation profiling on the Infinium EPIC platform.

**Results:**

From 2019 to 2023, 25 patients were enrolled based on initial pathology review, with 23 identified on 2nd review as grade 2 and 3 disease. 74% of patients received concurrent chemoradiotherapy with adjuvant chemotherapy. Of 9 patients with profiling, 5 were classified as mesenchymal subtype, while 4 matched to a variety of other phenotypes, including a novel F type GBM. Despite similar histological appearance corresponding to “lower grade glioma”, molGBM classified as IDH-wildtype mesenchymal had mOS of 15.7 months (95% CI 15.5-NA) while the other tumors had a mOS of 37.7 months (95% CI 10.9-NA).

**Conclusion:**

Our results demonstrate underlying heterogeneity within the molGBM population, pointing to future hypothesis-generating risk stratification strategies. We also demonstrate the feasibility of CTV reduction with therapy intensification to set a practice standard for RT management of non-enhancing molGBM.

**Supplementary Information:**

The online version contains supplementary material available at 10.1007/s11060-025-05269-6.

## Introduction

Evidence-based standard-of-care management principles for the newly termed molecular glioblastoma (molGBM) are lacking. molGBM is a subset of IDH-wildtype GBM not exhibiting the cardinal histopathological features of microvascular proliferation and/or necrosis historically associated with WHO grade 4 classification, and prior to the 2021 WHO Classification of CNS neoplasms (WHO 2021) was generally classified as “lower grade glioma” [1]. Moreover, molGBM with lower grade features exhibit minimal-to-no contrast enhancement on imaging with gadolinium-based contrast agents, distinct survival outcomes, and on-treatment radiation treatment (RT) imaging changes [2, 3].

Prior data suggesting that molGBM and histological GBM (histGBM) have similar outcomes were based on univariable analysis that did not adjust for the fact that molGBM patients were historically treated with less intense therapy [1, 4–7], including potentially RT without chemotherapy or doses as low as 45–54 Gy. While the median overall survival (OS) for histGBM (histological WHO grade 4) and molGBM (histological grade 2/3) were similar at less than 2 years, 3 year survival was estimated at less than 10% among 373 histGBMs and above 20% among 55 molGBMs [1]. A retrospective multi-center analysis of 65 molGBMs which did not differentiate the timing of radiation and chemotherapy found similar median OS to histGBM but higher progression-free survival (PFS) compared to histGBMs [2]. Other registry data not adjusting for therapy received, identified a median range OS for grade 3 vs. grade 2 astrocytomas of 8.8 to 21.5 months [8].

Importantly, prospective data on radiation volume techniques, imaging response and toxicity are lacking for this unique cohort of patients with potentially extended survival. Finally, differences in survival within the molGBM population suggest the potential for underlying heterogeneity. However, classifying metrics, molecular or otherwise, that effectively delineate the various components of molGBM have not been established, contributing to sub-optimal patient management. These knowledge gaps indicate a need for more rigorous analysis through prospective clinical trial evaluation.

Accordingly, the goals of our phase II trial were to evaluate outcomes for molGBM delivered treatment intensification in comparison to historical controls using tighter RT margins similar to lower grade gliomas while allowing concurrent and adjuvant chemotherapy. The study is currently ongoing for determining the final primary endpoint of PFS; hence here, we present exploratory endpoints related to DNA methylation-based stratification/epigenetic profiling correlated with OS. Our preliminary findings highlight a critical need to risk stratify molGBM based on current RT outcomes and biomolecular data. In the absence of this granularity, effectively establishing standard-of-care management principles for evidence-based treatment of the different molGBM subtypes will likely remain challenging.

## Methods

### Study design and participants

This is a single center single cohort phase II trial for molecular GBM/non-histological GBM. Patients ≥ 18 that prior to WHO 2021 would be IDH wildtype glioma, WHO grade 2 or 3 (historically termed diffuse astrocytoma, oligoastrocytoma, anaplastic astrocytoma, anaplastic oligoastrocytoma, or otherwise IDH wildtype gliomas) were eligible. Patients required a Karnofsky performance score (KPS) of at least 70 and had to be candidates for radiotherapy. Patients with multicentric disease, prior metastatic disease, and/or prior radiotherapy or chemotherapy for a brain tumor or leptomeningeal disease were excluded. Participants receive dose escalation from historical management to 60 Gy to the gross tumor volume (GTV) and 50 Gy to the clinical tumor volume (CTV). Concurrent oral temozolomide followed by adjuvant temozolomide after completion of radiation was allowed and at the discretion of treating physician to permit electing sequential therapy when appropriate. The protocol was approved by the institutional review board (ClinicalTrials.gov NCT04623931).

### Procedures

**Fig. 1:**
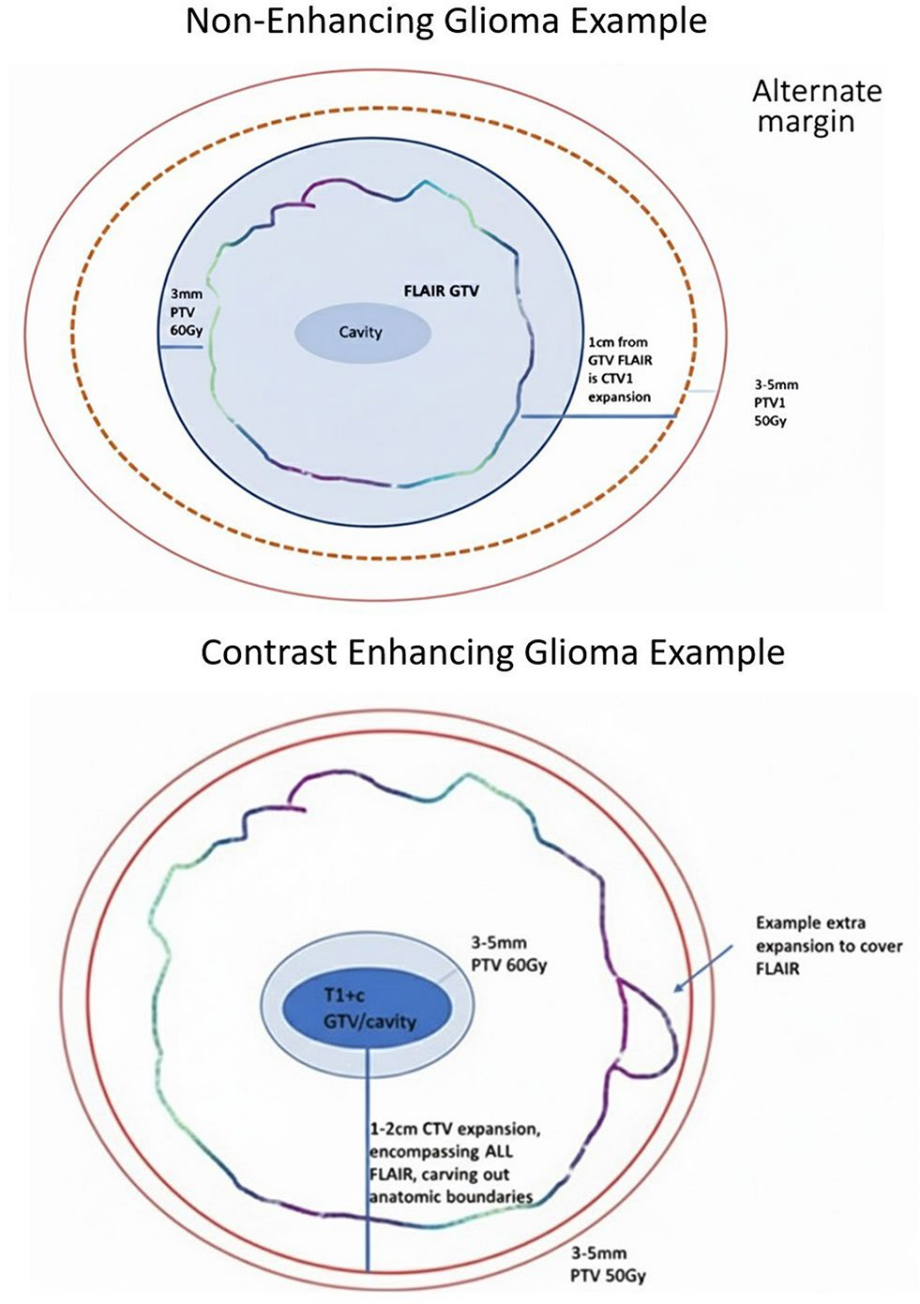
Protocol therapy volumes. (**A**) Non-enhancing glioma example &, (**B**) Contrast enhancing glioma example. *Approach of non-enhancing can also be used for enhancing tumor as long as addition CTV expansion beyond FLAIR edge is ≤ 1 cm

Patients were treated with radiotherapy using the following parameters- Gross tumor volume (GTV), including cavity and contrast enhancement and Clinical tumor volume (CTV) was defined as an expansion from GTV of 1–2 cm, modified to respect anatomical boundaries. For predominantly non-enhancing tumor, the margin was 1 cm from the GTV of FLAIR (fluid attenuation inversion recovery)-defined tumor. Consistent with the approach on protocol (Fig. 1A and B), FLAIR tumor extending beyond the uniform expansion from enhancing disease was covered by a non-geometric expansion. The CTV dose was 50 Gy in 30 fractions using a simultaneously integrated boost (SIB) technique. Planning tumor volume (PTV) 60 Gy in 30 fraction included a 3 mm expansion on GTV only, and PTV 50 Gy included a 3 mm expansion on CTV. Intensity modulated radiation therapy (IMRT)/ Volumetric modulated arc therapy (VMAT) or Protons could be utilized.

### Outcomes and statistical analysis

As an ongoing study, we present preliminary exploratory analyses, including correlative biomarker findings with OS as allowed by the protocol. The Kaplan-Meier method, with variance estimated using Greenwood’s formula, was used to estimate survival curves with survival/follow up censored to 2025. The restricted mean survival time (RMST) [9] over a 3-year period was also calculated. Between-group survival comparisons were performed using the log-rank test as well as the two-sample Wald test of RMST differences, with standard errors obtained via the delta method. Univariable and multivariable Cox proportional hazards models are implemented to explore potential risk factors. The pending final analysis will be conducted once the accrual goal of 38 evaluable of 40 total patients is reached which will yield a maximum half-width of 0.159 for the 95% normal-approximation confidence interval of the one-year PFS rate.

### IDH status/ next generation Sequencing/ DNA methylation/ epigenetic profiling

IDH status was screened by immunohistochemistry and tested for confirmation with next generation sequencing for all patients using Oncomine or MAPPs. Next, epigenetic profiling was an exploratory endpoint with limited remaining tissue. DNA methylation profiling was done on the Infinium EPIC platform by the MD Anderson Advanced Technology Genomics Core (ATGC) [10]. For degraded samples, further analysis was conducted using Illumina’s Infinium HD FFPE QC. Whole genome amplification, enzymatic fragmentation, and hybridization to Illumina Infinium Methylation EPICv2.0 was performed. A methylation class score had to be above the cutoff of 0.9 for a high-confidence match.

## Results

**Fig. 2:**
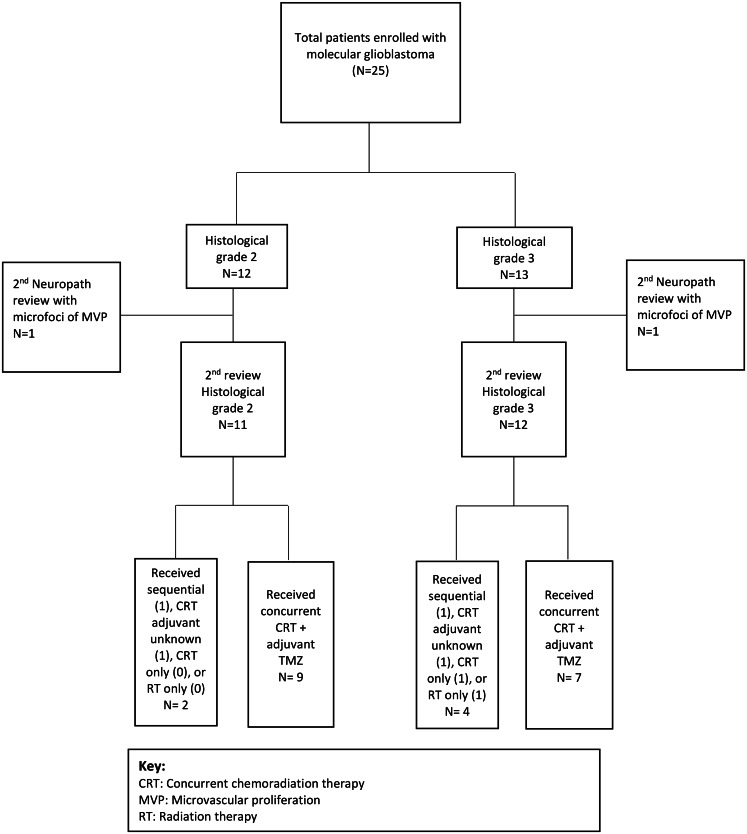
Consort diagram of clinical trial patients enrolled

From 2019 to 2023, 25 patients were enrolled on protocol (Fig. 2 CONSORT). All samples underwent a second study neuropathologist review and 2 patients initially diagnosed with lower grade disease were found to have microscopic foci of microvascular proliferation and thus reclassified as WHO grade 4. Age ranged from 21 to 74 years, with median age of 62 years (Table 1). Those with WHO grade 2 disease had median age of 64 compared to 60.5 for those with WHO grade 3. Patients had excellent KPS in general with 69% with scores of 90–100, and 17% with scores of 80. Though concurrent chemotherapy was at the discretion of the treating physician, adjuvant TMZ was planned initially for all patients. Four patients were coded unknown or not received due to COVID period impacting follow-up with limited data on the cycles known to be received locally or withdrawal (2), 1 deferred due to disease progression and unrelated thrombocytopenia and 1 RT alone due to clinical decline prior to sequential therapy. In summary, 74% of patients received concurrent chemoradiotherapy (CRT) with adjuvant chemotherapy and 9% received sequential RT followed by TMZ. Of those completing adjuvant TMZ, 9 completed 6–12 cycles and 10 completed 1–5 cycles.

**Table 1:** Trial patient characteristics and therapy

Characteristics	Patient (*N*)	%
**Total**	23	
**Sex**		
Male	12	52.2
Female	11	47.8
**Age** (years, median)	62	
Median age, histological grade 2	64	
Median age, histological grade 3	60.5	
18–39 years	3	13.0
40–59 years	7	30.4
60–69 years	10	43.5
≥70 years	3	13.0
**Race/Ethnicity**		
Non-Hispanic White	21	91.3
Non-Hispanic Black	1	4.3
Did not specify	1	4.3
**KPS**		
70–80	7	30.4
90–100	16	69.6
**Grade on 2nd neuropathology review**		
2	11	47.8
3	12	52.2
**RT Treatment**		
Concurrent chemoradiation (CRT), and adjuvant TMZ chemo	17	73.9
Sequential Radiation Therapy (RT), adjuvant TMZ chemo	2	8.7
Concurrent CRT received, adjuvant TMZ unknown	2	8.7
Concurrent CRT only with no adjuvant TMZ	1	4.3
Radiotherapy (RT) alone only	1	4.3
**Adjuvant chemo cycles**		
6–12 cycles TMZ	9	39.1
4–5 cycles TMZ	5	21.7
1–3 cycles TMZ	5	21.7
No adjuvant or Unknown	4	17.4
**Epigenetic Profile**		
Mesenchymal GBM phenotype	5	21.7
Non-mesenchymal GBM phenotype	4	17.4

All patients received the intended prescription dose of 60 Gy (Supplement Table A). For the majority of molGBM cases, the cavity with FLAIR tumor comprised the GTV followed by a CTV expansion of 1 cm with preoperative non-enhancing disease. For the 7 cases of molGBM with minimal preoperative enhancement, GTV was defined as the cavity plus enhancing disease and the CTV expansion included an expansion of 1.5–2 cm, respecting anatomical boundaries. No patients required a second CTV (i.e. CTV2). GTV median volume was 70.1 cc and CTV median volume was 216.1 cc. Asymptomatic radiographic radiation necrosis/treatment effect changes were noted in 5 cases ranging in date of identification from 1 to 21 months post RT. An episode of cytopenia during RT was only noted among patients receiving concurrent chemotherapy, with a total of 6 patients (26%) developing either lymphopenia (5), anemia (3), or leukopenia (2). Grade 3 or higher lymphopenia was only noted in 2 cases (8.7%). After RT and during adjuvant phase, an episode of any cytopenia was noted in 10 patients.

NGS testing and molecular features are presented in Supplemental Table B. From NGS testing, EGFR amplifications (gene copy number variation) were present in 5 of 22 patients, TERT mutations in 14 patients, and of those with additional chromosomal microarray analysis with both chromosomal gain of 7/ loss of 10 in 8 patients. Furthermore, FGFR3/TACC3 fusions were present in 3 patients, CDKN2A/2B homozygous deletion in 1 patient, and a H3-3 A G34/G35 mutation in 1 patient. DNA methylation data and quality assurance sampling analysis was available for 9 patients. This analysis demonstrated that 5 molGBMs were classified as IDH-wildtype GBM, mesenchymal and 4 as some other entity. These alternative classifications included: Adult-type diffuse high grade glioma IDH-wildtype subtype F-novel (1), control pons tissue (1), H3 G34-mutant diffuse hemispheric glioma (1), and ganglioglioma (1). Amongst cases classified as IDH-wildtype GBM, mesenchymal, three were histological WHO grade 2 cases and two were histological WHO grade 3. Among those with other DNA methylation-based classifications, three were classified as histological WHO grade 2 and one as WHO grade 3. From this cohort, 7 patients had unmethylated MGMT tumors and 2 methylated MGMT tumors.

**Fig. 3:**
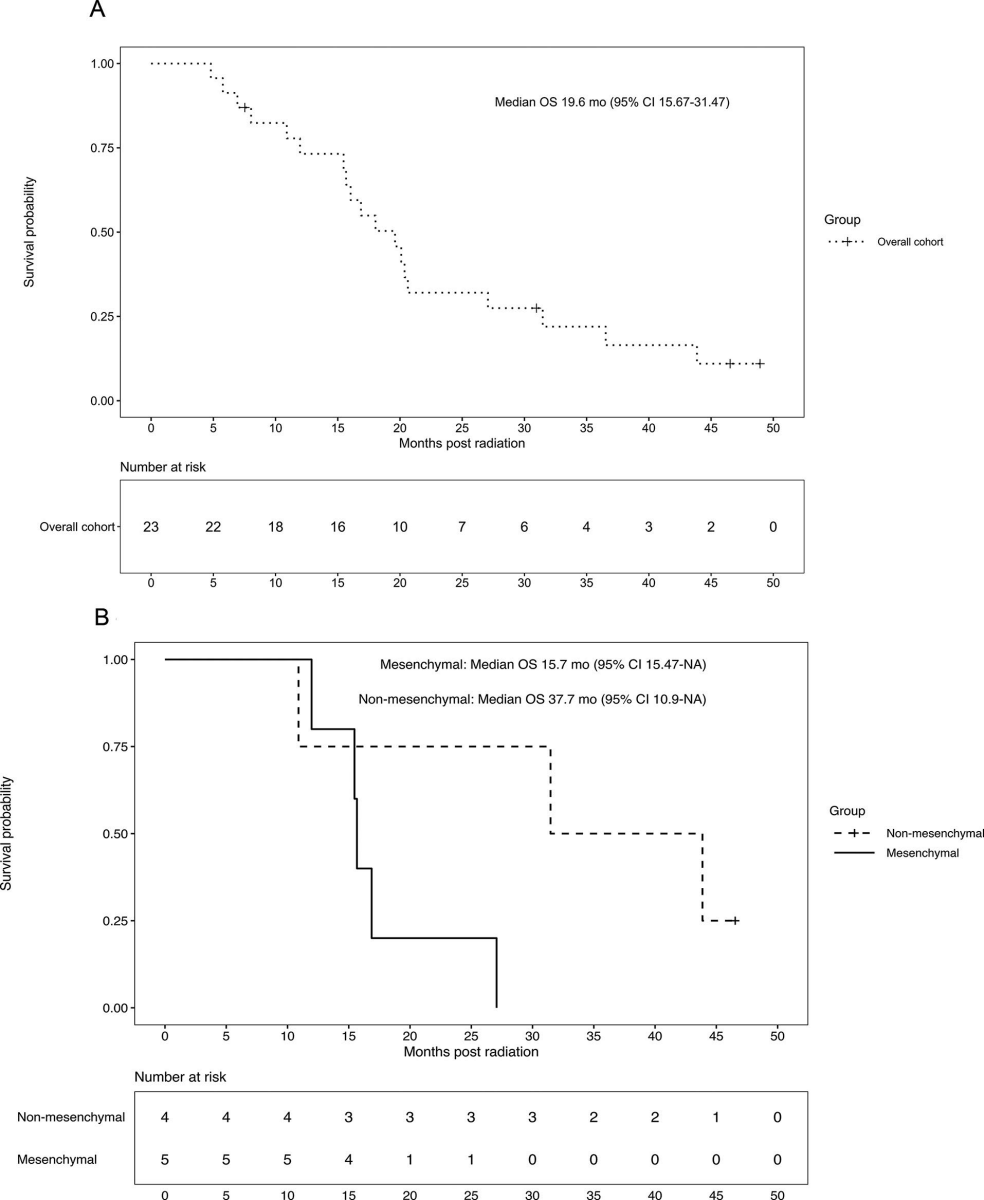
Overall Survival by Total Cohort (**A**) and Subset by Epigenetic stratification (molecular GBM mesenchymal vs non-mesenchymal) (**B**)

The median follow-up period was approximately 46.5 months and median OS was 19.6 months (95% CI 15.7–31.5) for the 23 patients (Fig. 3A). Stratifying by the available DNA methylation demonstrated that, despite similar histopathology across our molGBM cohort, OS was 15.7 months (95% CI 15.5-NA) for IDH-wildtype GBM, mesenchymal and 37.7 months (95% CI 10.9-NA) for otherwise classified cases (Fig. 3B). The 3-year RMSTs for the mesenchymal-DNA and non-mesenchymal groups were 17.4 months (95% CI, 12.9–21.9) and 28.6 months (95% CI, 18.4–38.8), respectively. The difference in 3-year RMST between the two groups was − 11.2 months (95% CI, − 22.3 to 0.1; *p* = 0.048).

For the overall 23 patient cohort, median OS for WHO Grade 2 was 27.1 months (95% CI 16-NA) and for WHO Grade 3 patients 17.6 months (95% CI 10.9-NA). WHO grade 2 IDH-wildtype GBM, mesenchymal cases had a wider range in OS (15.5 months – 27.1 months) compared to WHO grade 3 counterparts (12 months − 15.7 months). Multivariable analysis of OS adjusting for age, KPS, grade and treatment did not reach statistical significance (Table 2). Treatment of concurrent chemoradiation vs. sequential therapy was not statistically significant, though analysis was not powered to analyze differences in therapy specifically.

**Table 2:** Univariable and multivariable Cox proportional hazard models of overall survival

Characteristics		Univariable Cox			Multivariable Cox		
	N	HR	CI	p-value	HR	CI	*p*-value
**Total**	23						
**Profile**							
GBM Mesenchymal (Reference)	5						
GBM Non-mesenchymal	4	0.255	(0.054, 1.200)	0.084	0.251	(0.035, 1.832)	0.173
Unavailable	14	0.569	(0.189, 1.713)	0.316	0.638	(0.133, 3.050)	0.573
**Age**		0.527	(0.306, 0.907)	0.021	0.617	(0.284, 1.341)	0.223
**KPS**							
70–80 (Reference)	7						
90–100	16	1.419	(0.499, 4.041)	0.512	1.360	(0.411, 4.503)	0.614
**Grade on 2nd neuropathology review**							
2 (reference)	11						
3	12	1.652	(0.659, 4.143)	0.284	0.824	(0.232, 2.929)	0.765
**RT Treatment**							
Concurrent chemoradiation (CRT), and adjuvant TMZ chemo (Reference)	17						
Sequential Radiation Therapy (RT), adjuvant TMZ chemo	2	1.042	(0.229, 4.732)	0.958	0.788	(0.138, 4.512)	0.789
Other or RT alone	4	10.671	(2.025, 56.230)	0.005	3.403	(0.371, 31.254)	0.279

## Discussion

Preliminary exploratory analysis of our trial found that outcomes for histological WHO grade 2/3 molGBM stratify by epigenetic profile reveals a doubling of OS for patients not classified as IDH-wildtype GBM, mesenchymal. Moreover, we detected within our cohort rarer glioma subtypes with limited prior reporting of outcome measures. Importantly these patients received treatment intensification in either dose to 60 Gy or use of concurrent temozolomide, compared to the less aggressive treatment regimens historically received. For instance, a prior phase II treatment intensification study of high-risk low-grade gliomas (RTOG 0424) utilized concurrent chemoradiation with 54 Gy [11] for having 3 out of 5 clinical presentations and predated the current molecular classification system. Lastly, this study did not include histological WHO grade 3 gliomas or IDH classification, which our protocol evaluates.

While community practice may consider empiric use of concurrent chemoradiation for molGBM, a clinical protocol is necessary for clinical implementation and to establish a standard-of-care approach for RT. Prospective data to evaluate the role of RT dose, RT volume margins, and future sequence of chemotherapy is necessary given the wide diversity in this unique subset of patients. There is not yet evidence to suggest that these 2 entities, histGBM versus molGBM, behave similarly with dose modification given the differences in their radiographic and epigenetic patterns. A randomized study of IDH wildtype grade 2/3 of CRT vs. RT alone identified a longer survival with CRT [12]. Next a post hoc retrospective analysis of CATNON [13], of the few newly classified molGBMs within the subcohort did not identify a benefit for CRT over sequential but importantly acknowledged that a prospective clinical trial is needed to appropriately address the question [14]. The CATNON study differs from our current prospective cohort because (1) CATNON was limited to histological WHO grade 3 gliomas, (2) it included type II gliomas (IDH mutants without 1p19q co-deletion), (3) it dosed patients to 59.4 Gy, with and without concurrent temozolomide, and (4) initially, it was not specifically designed for the IDH-wildtype molecular group. Also of note, post hoc retrospective analysis of the CATNON trial identified only 37 patients who met the new diagnostic criteria for molGBM receiving concurrent chemoradiotherapy out of 751 total patients with histological WHO grade 3 disease over >10 years [14]. These distinctions underscore the significance of the current trial at MDACC, as we have prospectively evaluated more than 20 patients in 1/3rd of the time it took to conduct the larger international CATNON study. Moreover, moving forward the prospective analysis will address both histological WHO grade 2 and 3 tumors, and may therefore identify different outcomes from the retrospective analysis.

Our protocol specifically provides highly meaningful clinical data on molGBM patient cohort when utilizing radiation therapy to 60 Gy with disease-customized margins and treatment planning. One of the challenges for therapy for these patients from a radiation delivery perspective has been the question of treatment planning margin creation for non-enhancing or minimally enhancing disease. For instance, patients with histological WHO grade 2–3 gliomas in the past could have been treated with radiation margins as low as 1 cm compared to a histGBMs with margins of 2 cm. This volumetrically increases the range of normal appearing brain by several magnitudes. Our protocol defines a uniform approach with GTV only to 60 Gy (including just a PTV to the 60 Gy volume), with 1 cm margin for CTV to 50 Gy. In contrast to reports of 44% cytopenia for histological GBM receiving CRT [15], the percentage of patients developing cytopenia during radiation was 26% on protocol, and only 30% if analyzing just among those receiving concurrent chemoradiation therapy. Next while the literature for standard RTOG volumes identifies grade 3 + lymphopenia as 15% [16], our analysis noted only 8.7%. Radionecrosis was 22%, which was lower than the rates of 34% reported from RTOG volumes. Prior data from MDACC has demonstrated that use of smaller volumes both decreases the likelihood of radionecrosis and lymphopenia [16], though that study was done in histGBM with CTV margin of 2 cm and compared to RTOG volumes with 2 CTV expansions. Importantly, our presented data for specifically molGBM demonstrates feasibility for further reduction to 1 cm with this single CTV, which can be additionally customized by DNA methylation-based classification in the future.

Our trial results are hypothesis generating and suggest that there is an opportunity to improve stratification of molGBM with high dimensional epigenetic data that unlocks the potential to differentiate more favorable outcomes of molGBM from true histGBM. DNA methylation classification of tumors utilizes a reference tumor cohort to generate genome-wide DNA methylation profiles [10, 17–25]. While a notable study explored mean global DNA methylation profiles of IDH wildtype gliomas as a prognostic marker [26], importantly, the study design did not specifically examine the differential profile of histological appearing grade 2/3 molGBM. In histGBM, DNA methylations profiling has identified that those with GBM mesenchymal profile have worse outcomes compared to proneural, neural and classical [27]. Our data for specifically molGBM identifies similarly poor prognosis for the mesenchymal profile, and uniquely identifies a high range of diversity within the cohort of non-mesenchymal GBM profiles.

Highlighting our population’s rarity, a retrospective study of lower grade-appearing gliomas with DNA methylation profiling from 2007 to 2016 identified only 3% of grade 2 and 4% of grade 3 gliomas as IDH wildtype. When comparing the larger cohort of >90% IDH mutant patients, the IDH wildtype cohort were the most heterogeneous molecular group by epigenetic profile. In fact, epigenetic profiling provided more comprehensive data for detection of rarer entities that could easily be misclassified with conventional techniques, particularly when differentiating the more indolent IDH wildtype gliomas from other molGBM [28]. For instance, 2 of the non-mesenchymal tumors are prominent in pediatric gliomas, and thus the presentation in adults highlights heterogeneity representing important challenges to overcome in the literature and for clinicians to be aware of. Future studies could potentially improve on this to identify novel entities and/or tailor therapy prospectively.

### Limitations

While our prospective trial provides valuable insights, it is limited by sample size due to the rarity of molGBM as a disease subtype. There was also limited tissue availability for analysis in the cohort for epigenetic classification. We acknowledge that, in a small cohort, a multivariable Cox regression model is susceptible to overfitting. For this reason, we present this model as an exploratory analysis to inform future investigations. Nonetheless, in the smaller subset our data suggests a trend toward a survival difference between non-mesenchymal and mesenchymal GBM, with their 3-year RMSTs showing a statistically significant difference. Our study was not powered to compare outcomes of patients receiving concurrent chemoradiation therapy to those receiving sequential therapy. Such analyses will likely require a prospective randomized controlled multi-center study. Lastly at the time the protocol opened in 2019, it included all IDH wildtype gliomas that did not have histological grade 4 features and since then some subgroups such as a H3G34 mutant case are defined. Nevertheless, our data suggest that beyond histological WHO grade, the incorporation of additional molecular classification metrics, specifically including epigenetic/ DNA methylation profiling, will be important for clinical stratification. Completion of the trial will provide a foundational dataset for comparison to historical controls/retrospective multicenter studies and reveal cases not yet identified that may become distinct in future generations. Lastly, the long-term toxicity and neuro-cognitive metrics can only be substantively analyzed at study completion.

## Conclusions

Our preliminary results show that epigenetic profiling may hold higher significance for molGBM patients for improved risk stratification and management. It also demonstrates the feasibility of radiation CTV margin reduction in treatment intensification, based on lower rates of cytopenia, lymphopenia and radiation necrosis. Identifying molGBM with non-mesenchymal GBM epigenetic profile have differential outcomes compared to mesenchymal molGBM is hypothesis generating for future therapeutic studies, and suggests clinicians explore further testing beyond NGS testing to best classify this spectrum of gliomas.

## Electronic supplementary material

Below is the link to the electronic supplementary material.

Supplementary Material 1

## Data Availability

Data is provided within the manuscript or supplementary information files. As an ongoing trial, primary data would not be shared until study closure and also primary and exploratory analysis are reported.
