# Supplementary material for: Prospective phase II clinical trial of molecular glioblastoma (historical grade 2 and 3 IDH wildtype gliomas) preliminary novel exploratory analyses: Treatment intensification, margin reduction and epigenetic stratified outcomes with radiation therapy and chemotherapy
Source: J Neurooncol. 2025 Nov 5;176(1):72. doi: 10.1007/s11060-025-05269-6 (PMC12589214; doi:10.1007/s11060-025-05269-6)
Supplement: Supplementary file 1 — Supplementary Material 1 [file 11060_2025_5269_MOESM1_ESM.docx]

**Supplement Table A:** Radiation Volumes and Therapy

| **Number of patients** | **GTV volume (cc)** | **CTV**  **Volume (cc)** | **CTV1**  **margin** | **GTV dose (cGy)** | **CTV dose**  **(cGy)** | **GTV D99 (cGy)** | **CTV1 D99 (cGy)** |
| --- | --- | --- | --- | --- | --- | --- | --- |
| 1 | 36.5 | 97 | GTV+2CM | 6000 | 5000 | 5761 | 5087 |
| 2 | 66.87 | 211.96 | GTV+2CM | 6000 | 5000 | 6082 | 5109 |
| 3 | 24.2 | 244 | GTV+2CM | 6000 | 5000 | 5563 | 5055 |
| 4 | 86.6 | 305 | GTV+1.5CM | 6000 | 5000 | 6154 | 5135 |
| 5 | 43.7 | 155 | GTV + 1CM | 6000 | 5000 | 6136 | 5198 |
| 6 | 63.1 | 154 | GTV + 1 CM | 6000 | 5000 | 5574 | 5245 |
| 7 | 47.5 | 151 | GTV + 1 CM | 6000 | 5000 | 6059 | 5220 |
| 8 | 122 | 315 | GTV + 1 CM | 6000 | 5000 | 5951 | 5236 |
| 9 | 43.4 | 139 | GTV + 1 CM | 6000 | 5000 | 5896 | 5336 |
| 10 | 42.4 | 106 | GTV + 1CM | 6000 | 5000 | 5653 | 5085 |
| 11 | 35.5 | 160 | GTV + 1 CM | 6000 | 5000 | 6068 | 5208 |
| 12 | 184 | 447 | GTV + 1.5CM | 6000 | 5000 | 5337 | 5115 |
| 13 | 56.6 | 167 | GTV + 1CM | 6000 | 5000 | 6132 | 5430 |
| 14 | 131 | 296 | GTV + 1CM | 6000 | 5000 | 5873 | 5873 |
| 15 | 49.8 | 154 | GTV + 1CM | 6000 | 5000 | 6091 | 5256 |
| 16 | 77.1 | 226 | GTV + 1CM | 6000 | 5000 | 6100 | 5395 |
| 17 | 65.8 | 184 | GTV + 1CM | 6000 | 5000 | 6083 | 5286 |
| 18 | 63 | 199 | GTV + 1CM | 6000 | 5000 | 6092 | 5471 |
| 19 | 83.2 | 272 | GTV + 1CM | 6000 | 5000 | 6077 | 5271 |
| 20 | 46.9 | 169 | GTV + 1CM | 6000 | 5000 | 6006 | 5213 |
| 21 | 165 | 370 | GTV + 2CM | 6000 | 5000 | 5835 | 5075 |
| 22 | 22.2 | 72.2 | GTV+1CM | 6000 | 5000 | 6145 | 5351 |
| 23 | 56.3 | 376 | GTV+1.5cm | 6000 | 5000 | 6025 | 5183 |
| **Mean volume (cc)** | 70.1 | 216.1 |  |  |  |  |  |

*CTV expansion to cover all FLAIR tumor as required per protocol notes tumor up to 3cm from enhancement with appropriately encompassed non-geometric expansion.

**Supplement Table B:** Next Generation Sequencing Molecular Information

| **Patients** | **Gene Mutation**  **(Single Nucleotide Variant)** | **Gene**  **Amplification (Copy Number Variation)** | **Gene**  **Deletion**  **(Copy Number Variation)** | **Gene Fusion** | **Chromosome Gain of 7** | **Chromosome Loss of 10** |
| --- | --- | --- | --- | --- | --- | --- |
| 1 | EGFR, TERT, SETD2 |  |  |  | **Yes** | **Yes** |
| 2 | EGFR, PTEN, TERT, CREBBP |  |  |  |  | Yes |
| 3 | TERT | CDK4, EGFR, MDM2 |  |  |  |  |
| 4 | EGFR | EGFR, TERT |  |  |  |  |
| 5 | PTEN |  |  |  | **Yes** | **Yes** |
| 6 | PALB2 |  |  |  | **Yes** | **Yes** |
| 7 | EGFR, TERT | MDM4 |  |  |  |  |
| 8 | TERT |  |  | FGFR3/TACC3 |  | Yes |
| 9 | EGFR, PTEN, NF1 | CDK4 |  |  |  |  |
| 10 | POLE, EGFR, ATR, BRCA2 |  |  |  |  |  |
| 11 | NF1, TERT, PTEN, ATRX |  |  | FGFR3/TACC3 |  |  |
| 12 | TP53, H3-3A G34/G35 mutant [c.103G>A p.G35R (legacy G34R)] |  |  |  |  |  |
| 13 | PTEN, TERT, PIK3R1, STAG2, BCOR |  |  |  |  |  |
| 14 | PIK3R1 x2 (mutations), TERT |  |  |  |  |  |
| 15 | EGFR, TERT | EGFR |  |  | **Yes** | **Yes** |
| 16 | NF1, PTEN, RICTOR, TERT, YAP1 |  |  |  |  |  |
| 17 | AURKB, SETBP1, TERT | FRS2, MDM2, PTPRB |  |  | **Yes** | **Yes** |
| 18 | Inadequate specimen remaining |  |  |  |  |  |
| 19 | TERT |  |  |  | **Yes** | **Yes** |
| 20 | CDH1, EGFR x2 (mutations), FLT3, GRM3, TERT, PIK3R1 | EGFR |  |  | **Yes** | **Yes** |
| 21 | TERT |  | PTEN(10q23)/CEP 10 |  | **Yes** | **Yes** |
| 22 | EGFR, GRM3, TERT, TSC1 |  |  | FGFR3/TACC3 | Yes |  |
| 23 | ATRX, PTEN, TP53 | EGFR | CDKN2A, CDKN2B |  |  |  |
